# Human Alphacoronavirus Universal Primers for Genome Amplification and Sequencing

**DOI:** 10.3389/fmicb.2022.789665

**Published:** 2022-03-25

**Authors:** Sungmi Choi, Kwan Woo Kim, Keun Bon Ku, Seong-Jun Kim, Changwoo Park, Dongju Park, Seil Kim, Hana Yi

**Affiliations:** ^1^Interdisciplinary Program in Precision Public Health, Korea University, Seoul, South Korea; ^2^Center for Convergent Research of Emerging Virus Infection, Korea Research Institute of Chemical Technology, Daejeon, South Korea; ^3^Microbiological Analysis Team, Group for Biometrology, Korea Research Institute of Standards and Science (KRISS), Daejeon, South Korea; ^4^Department of Agricultural Biotechnology, Seoul National University, Seoul, South Korea; ^5^Department of Biological Science, Chungnam National University, Daejeon, South Korea; ^6^Department of Bio-Analysis Science, University of Science and Technology, Daejeon, South Korea; ^7^School of Biosystems and Biomedical Sciences, Korea University, Seoul, South Korea

**Keywords:** alphacoronavirus, genome amplification, genome sequencing, primer, diagnosis

## Abstract

Rapid and accurate sequencing covering the entire genome is essential to identify genetic variations of viral pathogens. However, due to the low viral titers in clinical samples, certain amplification steps are required for viral genome sequencing. At present, there are no universal primers available for alphacoronaviruses and that, since these viruses have diverse strains, new primers specific to the target strain must be continuously developed for sequencing. Thus, in this study, we aimed to develop a universal primer set valid for all human alphacoronaviruses and applicable to samples containing trace amounts of the virus. To this aim, we designed overlapping primer pairs capable of amplifying the entire genome of all known human alphacoronaviruses. The selected primers, named the AC primer set, were composed of 10 primer pairs stretching over the entire genome of alphacoronaviruses, and produced PCR products of the expected size (3–5 kb) from both the HCoV-229E and HCoV-NL63 strains. After genome amplification, an evaluation using various sequencing platforms was carried out. The amplicon library sequencing data were assembled into complete genome sequences in all sequencing strategies examined in this study. The sequencing accuracy varied depending on the sequencing technology, but all sequencing methods showed a sequencing error of less than 0.01%. In the mock clinical specimen, the detection limit was 10^−3^ PFU/ml (10^2^ copies/ml). The AC primer set and experimental procedure optimized in this study may enable the fast diagnosis of mutant alphacoronaviruses in future epidemics.

## Introduction

Coronaviruses have had a major impact on mankind since the mid-1960s. They are classified into four distinct genera: alpha, beta, gamma, and delta. Both alpha- and beta- coronaviruses are known to cause diseases in humans. Unlike betacoronaviruses, which include deadly species such as MERS-CoV and SARS-CoV, alphacoronaviruses have received relatively little attention due to their low fatality rate (Vassilara et al., 2018). Two types of alphacoronaviruses infect humans: HCoV-229E, which was first described in the mid-1960s, and HCoV-NL63, which was identified in 2004. These two types of alphacoronaviruses are known to cause 30% of common colds in humans (Su et al., 2016).

Alphacoronaviruses are diagnosed using real-time reverse transcription-polymerase chain reaction (rRT-PCR). ORF1a and b (van der Hoek et al., 2005), and nucleocapsid genes (Bergner et al., 2020; Morehouse et al., 2020) are used as diagnostic markers. However, false-negative results may occur when there is a mutation in the marker gene region (Kevadiya et al., 2021). Moreover, any viral variant located beside the short marker gene region cannot be determined through the conventional standard rRT-PCR. Thus, whole-genome sequencing is essential to determine whether a virus is a mutant strain (Gilchrist et al., 2015; World Health Organization, 2020). The use of whole genome sequencing to identify SARS-CoV-2 mutants is a good example (Wang et al., 2020; Zhou et al., 2020; Hu et al., 2021).

Since the viral load in clinical specimens is minimal, it is difficult to directly recover a sufficient amount of nucleic acid for genome sequencing. To solve this problem, various alphacoronavirus genome sequencing methods have been developed over the past 20 years. The first genome of HCoV-229E, reported in 2001, was sequenced by synthesizing it as cDNA and cloning it into the vaccinia virus (Thiel et al., 2001). In 2004, the complete genome of HCoV-NL63 was sequenced using the Virus-Discovery-cDNA AFLP (VIDISCA) method (van der Hoek et al., 2005). Currently, a method for amplifying a genome fragment using a strain-specific primer set is being used (Geng et al., 2012; Zhang et al., 2020). However, since coronaviruses have many mutants, new primers specific to the target strain must be continuously developed for sequencing.

In contrast, universal primers, which can be used for a wide range of variants within a viral species or a viral genus, have been developed for other viral groups. For example, universal primers applicable to all strains of the Ebola virus have been successfully designed and applied for epidemic surveillance (Quick et al., 2016). A universal primer set applicable to the entire betacoronavirus clade C, including the MERS coronavirus, has also been reported (Kim et al., 2021). In the case of the current COVID-19 pandemic, the ARTIC Network developed a primer panel and a multiplexed amplicon-based whole-viral-genome sequencing protocol, which enabled the rapid and accurate diagnosis of SARS-CoV-2 variants (Tyson et al., 2020).

Here, we aimed to develop a primer set and a sequencing method that can be universally applied to all human-infecting alphacoronaviruses. To that aim, a universal primer set, named AC primer set, was designed to secure sufficient nucleic acid levels for genome sequencing from a trace amount of virus. After genome amplification using the developed primers, an evaluation using various sequencing platforms was carried out.

## Materials and Methods

### Virus Cultivation and Ribonucleic Acid Extraction

The viruses HCoV-229E (ATCC VR-740) and HCoV-NL63 (NCCP 43214) were obtained from American Type Culture Collection and National Culture Collection for Pathogens, respectively. Viruses were cultivated using Vero E6 cells in a biosafety level 3 (BSL-3) laboratory at the Korea Research Institute of Chemical Technology (KRICT). Viral genomic RNAs were extracted using QIAmp Viral RNA Mini kit (Qiagen) according to the manufacturer’s instructions. The concentration of the extracted RNA was measured using digital PCR according to a method described previously (Park et al., 2021). The extracted RNA was stored at −80°C.

### Primer Design and Screening

For primer design, the complete genomes of 48 human alphacoronaviruses available at the Virus Pathogen Database and Analysis Resource (ViPR, Pickett et al., 2012)^1^ were collected (Accessed January 19, 2018). In addition to the human alphacoronaviruses, 10 complete genomes of animal-infected alphacoronaviruses with high similarity to HCoV-229E and HCoV-NL63 were collected. The sequences were aligned and primer design was performed using ViPR. The following criteria were considered for primer design: amplicon size 2–5 kb; melting temperatures 54°C–56°C; N base ≤2; primer length 18–22 bp, and inosine <2. The designed primer candidates were evaluated for their amplification performance using HCoV-229E cDNA as a PCR template. To select the best working primers, the size of amplicons, the range of overlaps between adjacent amplicons, the band singularity and intensity in gel electrophoresis, and the coverage of the genome region were considered.

### Evaluation of the Universality and Specificity of the Primers

To determine the specificity of the primers, the primer sequences were searched using Basic Local Alignment Search Tool (BLASTn) in the Human Genome and National Center for Biotechnology Information (NCBI) BLAST databases to examine whether similar sequences existed in genomes other than those of alphacoronaviruses. The versatility of the designed primers was evaluated *in silico* against the 124 human alphacoronavirus genome sequences available at ViPR (Accessed February 1, 2022) and 604 human alphacoronavirus gene sequences available at NCBI (Accessed February 1, 2022). The collected sequences and the designed primers were aligned with MEGA7 (Kumar et al., 2016) to count the number of mismatched nucleotides between the primer and genome sequences.

### RT

cDNAs were synthesized using SuperScript™ III First-Strand Synthesis SuperMix (Thermo Fisher Scientific) according to the protocol of the manufacturer. The initial reaction mixture (8 μl) contained 1 μl of total RNA, 1 μl of random hexamer (5′-NNNNNN-3′, 50 ng/μl), 1 μl of annealing buffer (10 mM), and 5 μl of RNase/DNase-free water. The mixture was incubated at 65°C for 5 min and then placed on ice for 1 min. Then, 10 μl of 2X First-Strand Reaction Mix and 2 μl of SuperScript™ III/RNaseOUT™ Enzyme Mix were further added to the reaction tube. The reaction mixture was incubated sequentially at 25°C for 10 min, 50°C for 90 min, and 85°C for 5 min. Three concentrations (50, 25, and 5 ng/rxn) of random hexamer solution were prepared and evaluated to optimize the concentration of the random primer required for RT. Three RT duration times were evaluated (50, 70, and 90 min).

### PCR and Purification

The PCR reaction mixture (50 μl) contained 1 μl of cDNA, 1 μl of forward primer (10 pmol/μl), 1 μl of reverse primer (10 pmol/μl), 25 μl of KAPA Hotstart Ready Mix (KAPA Biosystems), and 22 μl of distilled water. The PCR reaction was performed under the following conditions: initial denaturation at 98°C for 5 min; 30 cycles of denaturation at 98°C for 30 s, annealing at 55°C for 1 min, and extension at 72°C for 3 min; final extension at 72°C for 10 min. Then, the PCR product was stored at 4°C. To optimize the PCR conditions, different annealing temperatures (52°C, 55°C, and 58°C) were evaluated. The PCR products were purified using magnetic beads. The 10 purified amplicons were pooled into a single mixture for sequencing library construction.

### Sequencing and Assembly

#### RSII Sequencing

A sequencing library was constructed using 8 μg of amplicon mixture and a PacBio DNA Template Prep Kit 1.0 (Pacific Bioscience) according to the manufacturer’s standard procedure. The 20 kb sequencing library was sequenced using RSII (Pacific Bioscience). The sequencing reads were pre-assembled using the PreAssembler pipeline in the hierarchical genome assembly process (HGAP) of the SMRT Analysis v2.3.0 (Pacific Bioscience). Each contig was assembled from preassembled reads using Phred/Phrap/Consed version 27.0. Gaps between contigs were assembled through reference-guided assemble approaches based on the genome sequence of HCoV-229E (NC_002645). The genome alignments between contigs and NC_002645 were performed using NUCmer v.3.1. and the multiple alignments among sequences were carried out using MAUVE v.2.4.0.

#### MinION Sequencing

The sequencing library was constructed using 1 μg of amplicon mixture and a Ligation Sequencing Kit (Oxford Nanopore Technologies) according to the standard procedure of the manufacturer. Then, 1D amplicon sequencing was performed using MinION MK1B and R9.4.1 flow cell (Oxford Nanopore Technologies). Base-calling was performed for approximately 20 min using Guppy base-caller ver.3.4.4 + a296acb. Indexing and reference mapping were performed using bowtie2 software (2.2.9 v). Sequence alignment map and binary alignment map files were created using SAMtool v.1.1.0, and the variant site of the variant call format was checked using the BCF tool v.1.10.2v.

#### MiSeq Sequencing

A sequencing library was constructed using 100 ng of amplicon mixture and a TruSeq Nano DAN Kit (Illumina) according to the manufacturer’s standard procedure. The 301 bp paired-end sequencing was carried out using MiSeq (Illumina). The adapter sequences from the two paired-end files were removed with Cutadapt v1.11 + 13.g2af9c15 (Martin, 2011). The paired-end sequences were merged and assembled based on reference mapping using CLC workbench 9.5.3. (QIAGEN).

#### Sanger Sequencing

Individual amplicons were sequenced by the Sanger sequencing method. For this, primer walking was performed using additionally designed strain-specific primers (Supplementary Table 1). Sequencing was performed using an ABI 3730xl (Applied Biosystems). Sequences that satisfied the conditions of Phred score > 20 and length ≥ 700 bp were filtered and used for assembly. The filtered sequences were assembled using CodonCode Aligner v8.0.2 (CodonCode Corporation). The assembled single contig (27,273 bp) was further curated manually by observing the sequencing chromatograms.

#### Consensus Sequence

The consensus sequence was achieved by assembling the four genome sequences obtained through four different types of sequencing using CodonCode Aligner. This consensus sequence was used as a reference for calculating the accuracy and genome coverage of each sequencing platform.

### Phylogenetic Analysis

Multiple sequence alignment was performed using the cluster W algorithm embedded in MEGA 7.0.21. The 1–43 and > 27,270 nt positions (according to the reference genome sequence of 229E, NC0025405.1) were excluded from the genome tree analyses. For spike (S) protein amino acid tree inference, the 20,572–24,090 nt positions of the protein S coding gene were used. The neighbor-joining method and maximal likelihood method embedded in MEGA 7.0.21 were used for genome tree and S protein tree inference, respectively. The robustness of the trees was evaluated using the bootstrap analyses provided by MEGA. Based on the phylogenomic tree, 28 complete coronavirus genome sequences including 14 subgenera of alphacoronavirus encompassing human, bat, pig, alpaca, dog, and cat coronaviruses were selected as representative genome sequences. In addition, one genome sequence of betacoronavirus was included as an outgroup.

### Evaluation of Clinical Applicability

To evaluate the clinical applicability of the developed method, the mock clinical specimens were prepared by spiking cultured viral particles into synthetic human respiratory specimen. To mimic human nasal swab samples, one swab (about 10 mg) of Pooled Human Nasal Fluid (Innovative Research) was taken and dissolved in 1 ml transport media using 3 M™ Quick Swab (3 M). The cultured viral particles of HCoV-229E were serially diluted in the synthetic human respiratory specimens yielding viral concentrations of 10^−3^–10^0^ PFU/ml. RNA was extracted from mock clinical specimens using QIAamp Viral RNA Extraction kit according to the manufacturer’s instructions. The viral concentration in the extracted RNA was measured using the digital PCR. The reverse transcription, genome amplification, and MinION sequencing were performed as described above.

## Results

### Selection of Universal Primers for Alphacoronaviruses

**Figure 1:**
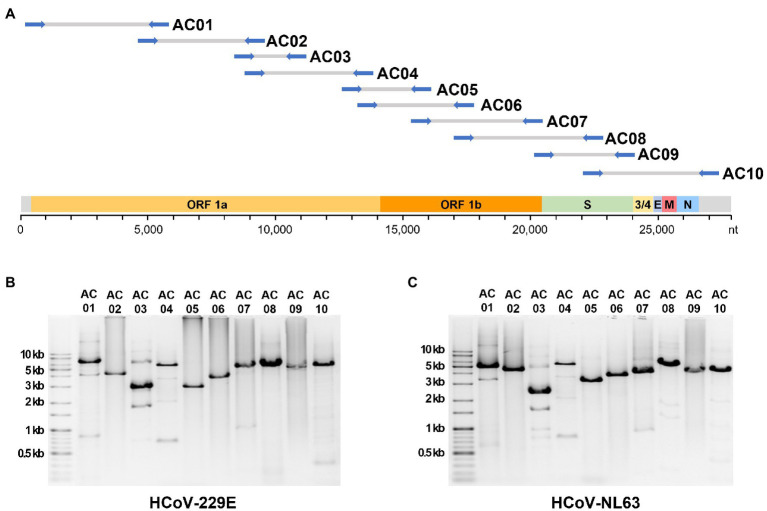
The localization of expected amplicons on the genome and the experimental amplification results. **(A)** The position of 10 primer sets alongside the genome sequence of human alphacoronaviruses. Gel electrophoresis image of PCR amplicons from the **(B)** 229E and **(C)** NL63 strains.

A total of 68 universal primer candidates were designed based on the sequences conserved in human alphacoronaviruses. PCR was performed using HCoV-229E cDNA as a template to select primers that produced a single band amplicon of the expected size in gel electrophoresis. The final selected 10 pairs of primers were named AC primer set, and each pair was designated as AC01–AC10 (Table 1). When the AC primer set was applied to the 229E and NL63 RNA, the expected amplicons were successfully produced for both strains (Figure 1). The average size of the 10 amplicons was 4,400 bp. The average overlap between the adjacent amplicons was 1,851 bp (i.e., 44.8% region of one amplicon was overlapped by the next following amplicons).

**Table 1:** List of human alphacoronavirus universal primers (AC primer set).

Primer name	Forward primer	Reverse primer	Length (bp)	Position (nt)
AC01	5´-TTTGTGTCTACTYYTCTCAACTA-3′	5´-GCATTMACCCAACAATTATTRT-3′	5,542	43–5,585
AC02	5´-TTTAATGTTGTWGGKCCICG-3´	5´-GCATAACAAGCRCARCGRTA-3´	4,719	4,358–9,077
AC03	5´-GGTGGTRAYAATGTTTATTGYTA-3´	5´-AGRCCAAAATCACTRTGYTTA-3´	2,912	8,201–11,113
AC04	5´-TAYCGYTGYGCTTGTTATGC-3´	5´-GGWACACCATCWATAAAMAC-3´	4,400	9,077–13,477
AC05	5´-CTGGTARYGGTCARGCTAT-3´	5´-CAYTTAGTRCACAACATMGG-3´	3,088	12,255–15,343
AC06	5´-GTKTTTATWGATGGTGTWCC-3´	5´-ACATCCATWCCYAACCAACC-3´	3,789	13,477–17,266
AC07	5´-CCKATGTTGTGYACTAARTG-3´	5´-CTRCCATCRTACATATCWGA-3´	4,704	15,343–20,047
AC08	5´-GGTTGGTTRGGWATGGATGT-3´	5´-TAAGGCRTCTTCWATRGTTTTACA-3´	5,191	17,266–22,457
AC09	5´-TATGGTGATGTKTCWAAAACTAC-3´	5´-TCRTAATAAGGAAGTTTAGTTGA-3´	4,733	19,333–24,066
AC10	5´-TGTAAAACYATWGAAGAYGCCTTA-3´	5´-AAAAATGGCTCTTCCATTGTTGGC-3´	4,883	22,457–27,374

BLASTn was performed on the NCBI database to verify whether the selected AC primers had similarities with other genomes, such as those of betacoronaviruses or the human genome. There were no cases of similarity (*E*-value <10^−5^) with genomes other than those of alphacoronaviruses. This confirmed that the AC primer set had a high specificity for human alphacoronaviruses.

### Optimization of the RT and PCR Conditions

The optimal concentration of random hexamer required for RT was evaluated. The results revealed that at 25–50 ng/rxn, the reaction occurred at its best. To synthesize long (5 kb) amplicons using the SuperScriptIII First-Strand Synthesis System, a long synthesis time of 70 or 90 min was required. When the duration of RT was reduced to 50 min, a 5 kb amplicon was not produced. From evaluating the PCR annealing temperature, 55°C was determined to be the optimal PCR temperature, as it provided the best amplification efficiency as detected by gel electrophoresis. The total experimental time required for RT (2 h), PCR (4 h), purification (0.5 h), and gel electrophoresis (0.5 h) was 7 h.

### Sequencing

The following sequencing statistics were obtained after sequencing 229E in four different ways and NL63 using MiSeq. RSII produced 1.9 Gb (299,617 reads) of raw data, and one linear contig (27,899 × sequencing depth) was obtained after assembly. MinION produced 8.23 Gb (161,307 reads) and one contig (3,555×). MiSeq produced 1.4–1.5 Gb (9,586,702–10,786,596 reads) and one contig (321–327×). Sanger sequencing and primer walking 97 specific primers produced 0.3 Mb (184 reads) and one contig (4.8×). Consensus sequences for 229E (27,316 bp, accession number MZ712010) and NL63 (27,504 bp, MZ682627) were obtained by compiling the results of all sequencing platforms. The two consensus sequences obtained in this study were 100 and 99.87% similar to the respective reference sequences (NC0025405.1 and NC005831.1), respectively.

### Comparison of Sequencing Platforms

To evaluate the performance of each sequencing platform, the sequences of contigs from each platform were compared against the consensus sequence. The analytical pipeline and performance of the sequencing platforms are summarized in Supplementary Figure 1. Contigs obtained from Sanger sequencing were identical to the consensus sequence, and contigs obtained from other high-throughput sequencing platforms showed similarities of 99.99%. Among the high-throughput sequencing platforms, MiSeq showed the highest accuracy with a difference of 2 nts among the 27,227 nts compared, followed by RSII and MinION, with a difference of 4 nts.

**Figure 2:**
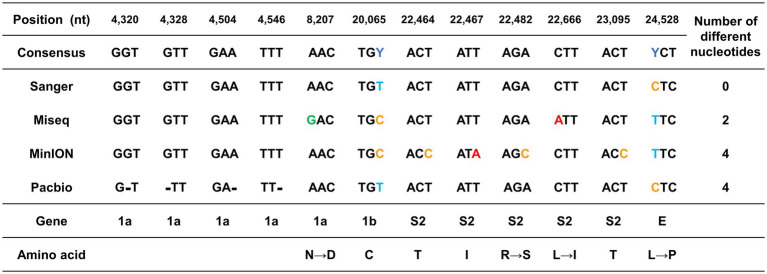
Graphical summary of the sequencing errors observed in this study. The genome sequences produced by each sequencing platform were compared against the consensus sequence. The nucleotide positions with sequence heterogeneity are marked in colored blocks. Dashes indicate gaps in the sequence of one genome relative to its counterpart.

Different sequences from the consensus sequence were found in the ORF1a, ORF1b, S2, and E genes at a total of 12 nt positions. The positions 20,065 bp of the ORF 1b and 24,588 bp of the E protein genes differed greatly depending on the sequencing platform (Figure 2). These two nts were sequenced differently as C or T, depending on the platform. The other 10 nt positions that were different from those in the consensus sequence differed in only one sequencing platform (Figure 2).

Since the mutation of the S protein is important for viral transmission and toxicity, the sequencing error that occurred in this part was further investigated. The 1 nt position in MiSeq and the 4 nt positions in MinION were errors that occurred in the S protein. All the sequencing errors observed both in MiSeq and MinION were translated into potential non-synonymous mutations. The error in MiSeq was an error due to a mutation in which leucine with a hydrophobic side chain was changed to isoleucine. The error in MinION was interpreted as a non-conservative missense mutation in which arginine with an electrically charged side chain was recognized as serine with a polar uncharged side chain.

Unlike substitution mutation errors in MiSeq or MinION, only nucleotide deletion errors were observed in RSII. Only four sequencing errors were found in the ORF 1a gene, which is a long gene that occupies more than 2/3 of the entire genome. Particularly, the errors were detected at 4,320–4,546 bp, which corresponded to the ends of the first and second amplicons.

### Phylogenetic Tree

**Figure 3:**
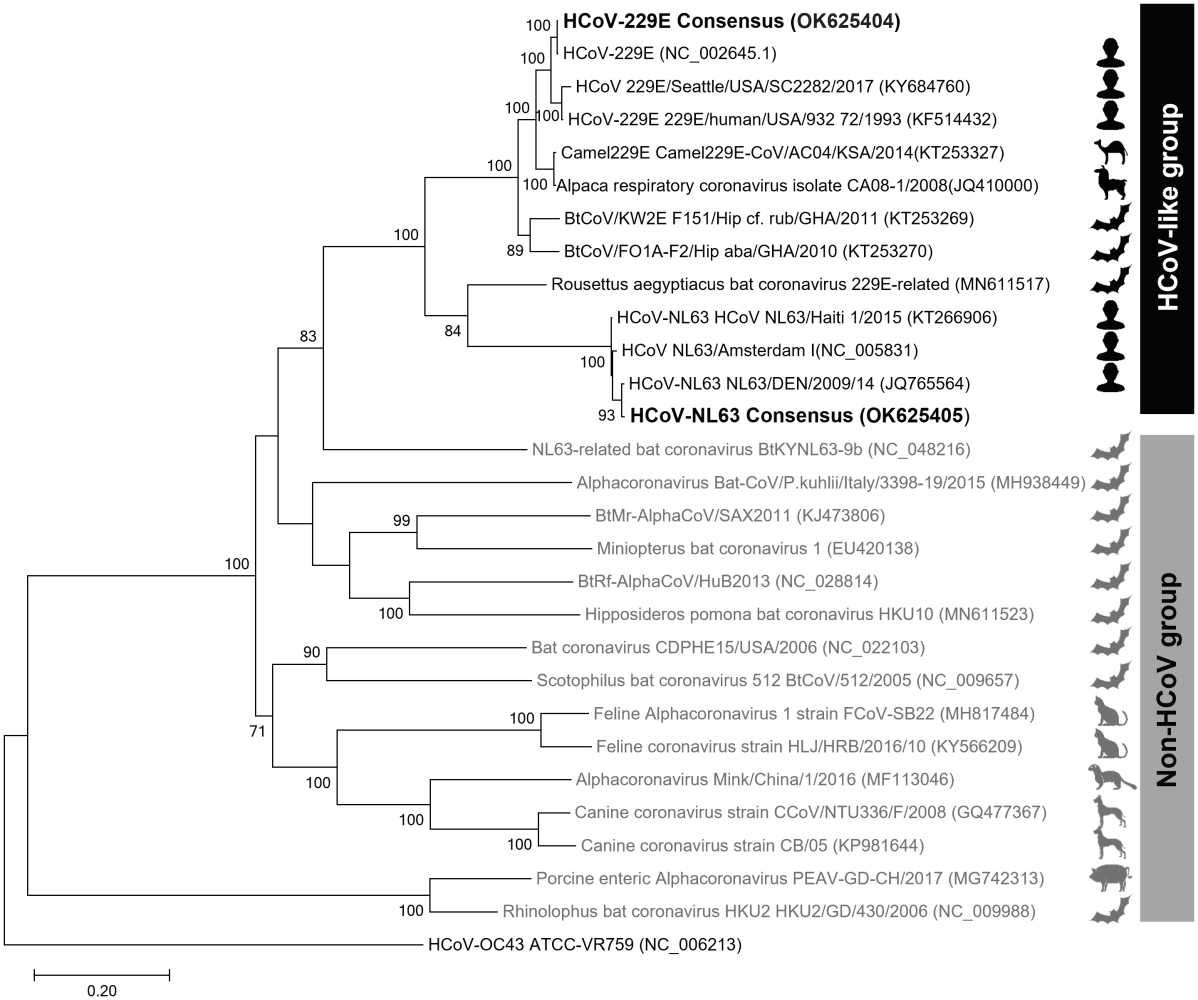
Spike protein amino acid sequence tree of alphacoronaviruses. The maximum-likelihood tree was inferred using the sequences at the 20,572–24,090 nt positions of the spike protein subunit1 (S1). The ingroups are the 28 representative sequences of 14 subgenera of alphacoronaviruses, and HCoV-OC43, a betacoronavirus, was used as an outgroup.

The phylogenetic trees created using the S protein and the whole-genome confirmed that the clade containing human alphacoronaviruses formed a clade independent of other non-human alphacoronaviruses (Figure 3; Supplementary Figure 2). In addition to human-infecting strains, the HCoV-like clade, to which the HCoV-229E and HCoV-NL63 belong, encompasses camel, alpaca, and bat coronaviruses. The intra-group similarities of the S protein within the HCoV-like clade were ≥64%, while the inter-group similarities between the HCoV-like group and the Non-HCoV group were less than 40%.

### Validation of Primer Universality

To evaluate the versatility of the AC primer set *in silico*, each primer was aligned with the representative genomes of the alphacoronavirus subgenera. The AC primers were well aligned to all alphacoronavirus genomes belonging to the HCoV-like clade. Mismatches of 0–2 nts were observed between individual primers and genomic sequences, and a mismatch of 0–11 nts was confirmed across all 20 primer sets (Supplementary Figure 3). All mismatches between the AC primers and the HCoV-like clade genomes were observed at locations other than the 3′ end, which is not known to significantly affect the PCR efficiency. In contrast, when compared with Non-HCoV genomes, 0–13 nt mismatches at the individual primer level and 36–91 nt mismatches at the entire 20 primer set were observed. Hence, the results showed that the AC primer set is universal for the HCoV-like clade but has low universality for the Non-HCoV group, which includes viruses that infect animals that are evolutionarily distant to humans.

To further evaluate the primer universality among the HCoV-like clade, 124 HCoV-229E and HCoV-NL63 genome sequences available at ViPR were examined. Most genomes belonging to the HCoV-like clade showed 0–2 primer mismatches in line with the result from the representative genomes (Supplementary Table 2). The five exceptional cases (4 bat and 1 camel coronaviruses) showed primer mismatches of 6–13, but those five strains have been known as non-HCoV group based on the S protein sequence phylogeny (Tao et al., 2017). As a result, the primer universality within the human alphacoronavirus was confirmed in a large dataset too.

To validate the primer universality against the protein gene sequences other than whole-genome sequences, 604 human alphacoronaviruses gene sequences available at NCBI were examined. Among the 604 protein gene sequences, only S gene sequences were in the range of target regions of AC primers. Primers AC08R and AC09R were located within the sequences of the S gene and showed 100% of identity against the 154 S gene sequences compared.

### Clinical Sample Applicability

When mock clinical samples were evaluated, target amplicons were produced even at the lowest viral concentration (10^−3^ PFU/ml). The amplicon mixtures from the lowest viral concentration were successfully sequenced using MinION and assembled into a complete genome sequence with a sequencing depth of 49×. We, therefore, decided 10^−3^ PFU/ml (10^2^ copies/ml) as the detection limit of this newly developed method in clinical samples.

## Discussion

Sufficient amounts of viral nucleic acid for genome sequencing are not easily obtained from clinical specimens. In the case of alphacoronaviruses, various genome sequencing methods have been developed to overcome this issue; however, current methods amplify a genome fragment using a strain-specific primer set. Since these viruses have diverse strains, new primers specific to the target strain must be continuously developed. In contrast, universal primers are applicable to a wide range of variants within a viral species or a viral genus and have been developed for other viral groups. Here, we sought to develop a primer set and a sequencing method that can be universally applied to all human-infecting alphacoronaviruses. In pursuing this aim, we designed a universal primer set and developed an optimal protocol that allows genome amplification and sequencing of all human alphacoronaviruses from a minimum viral concentration of 10^2^ copies/ml. The primer set was shown to be highly specific for human alphacoronaviruses and universal for the HCoV-like clade.

In PCR, the complementarity of the template and primer plays an important role in determining yield, and the mismatch is a cause of lower efficiency. Particularly, a mismatch at the 3′ end of the primer significantly increases the Ct compared to those in other positions and has the greatest influence on PCR failure (Kwok et al., 1990). All mismatches between the primers used in this study and the HCoV-like clade genome were observed at locations other than the 3′ end, suggesting that there will be no amplification problems using the AC primer set.

As a result of high-throughput sequencing, only 2–4 nts in the entire genome differed from the consensus sequence. The sequencing error rate observed in this study was not higher than the equipment error rate of sequencing platforms. Previous reports have reported sequencing error rates of <0.1% for PacBio CCS reads (Wenger et al., 2019), 0.1%–0.5% for Illumina MiSeq (Schirmer et al., 2016), and <1% for MinION (Sahlin and Medvedev, 2021). Another study reported that RSII is particularly prone to homopolymeric errors, and hence, (Amarasinghe et al., 2020). The results of this study also showed that the errors observed in RSII were all indel errors in homopolymeric regions, while the errors observed in MiSeq and MinION were non-synonymous mutations at random locations. This suggests that the sequencing errors observed in this study are an unavoidable problem that occurs due to the equipment and demonstrates that these are not errors caused by the AC primer set. To accurately diagnose viral mutations, it is necessary to overcome the equipment-induced errors by diversifying the sequencing platform for one sample or simultaneously sequencing multiple repeat samples on one sequencing platform.

Real-time and highly-precise genome sequencing is crucial for viral mutation surveillance. The Oxford Nanopore Technology platforms are superior to other sequencing platforms in the capacity of real-time sequencing, but inferior in base error rate as reported in other studies (Dohm et al., 2020; Delahaye and Nicolas, 2021; Wang et al., 2021). To solve this, the Oxford Nanopore Technology has adopted a reference-mapping-based genome assembly procedure rather than *de novo* assembly. The viral genome sequencing accuracy of MinION was reported as 99.98% in the study of SARS-CoV-2 genome amplicon sequencing from the clinical sample (Hourdel et al., 2020). We also obtained 99.99% accuracy demonstrating the feasibility of the MinION sequencer on viral diagnosis.

When Sanger sequencing results were automatically assembled using assembler software, a contig with a difference of 42 nts from the consensus was obtained. Contigs 100% identical to the reference sequence were obtained only after checking the sequencing chromatogram and performing manual curation. In this process, the following limitations using Sanger sequencing-based primer walking for viral genome sequencing were confirmed again. First, a large number of primers are required to cover the entire nucleotide sequence, as amplicons over 2 kb cannot be sequenced simultaneously. Second, the error rate is high because of insufficient sequencing depth. Third, since it requires a larger amount of RNA template compared to those of the other high-throughput sequencing platforms, it cannot be directly applied to clinical samples.

To amplify the full viral genome, methods such as sequence-independent single-primer amplification (SISPA), VIDISCA, and random primer RT-PCR are used. These methods are advantageous as they do not require group-specific primers and can randomly amplify various viral sequences (Sridhar et al., 2015). However, obtaining uniform sequencing coverage and depth across the entire genome by using the random amplification process is difficult (Karlsson et al., 2013). Additionally, since it is impossible to avoid amplification of host and viral nucleic acids together in the random amplification process, its application to clinical samples is difficult. Thus, to amplify and sequence the genome of human alphacoronaviruses, it is best to use universal primers that are specific to this group and cover all variants of this group.

With the recent development of high-throughput sequencing, viral genome sequences can be obtained through shotgun sequencing by omitting the viral nucleic acid amplification process. In a Peruvian bat study, two genomes were analyzed *via* shotgun sequencing of 10 pooled swab samples (Bergner et al., 2020). Since this method does not require primers, it is advantageous as it enables the identification and genome analysis of novel or highly mutated viruses. However, such kind of shotgun sequencing is not applicable to individual clinical samples. Unlike in environmental or animal samples, the viral titer in individual clinical samples is limited; therefore, it is not easy to assemble with sufficient coverage a viral genome through shotgun metagenome sequencing of clinical samples.

The biggest limitation of this study is that it has not been applied to actual clinical specimens. It is known that direct whole-genome sequencing analysis from clinical samples is difficult because of the various inhibitors present in clinical specimens. Thus, even if the method developed in this study yielded good results using viral RNAs, it should have been tested to confirm whether it is indeed applicable to clinical specimens. However, in a study on SARS-CoV-2, for which our research team is currently preparing a manuscript (data not shown), the universal primer-based amplification method was effective in clinical specimens and showed the same level of performance as *in vitro*. In addition, according to the amplicon-based sequencing protocol for the HCoV-OC43 strain, the primer panel worked successfully for clinical samples infected with the target virus (Nutho et al., 2020). The detection limit of the current method in mock clinical samples was as low as 10^2^ copies/ml which is similar to the known values reported in other studies of amplicon-based sequencing of clinical samples (Xiao et al., 2020; de Mello Malta et al., 2021). The LoD of our method is even equivalent to the qPCR method the value of which is known as 10–500 copies/ml (Fung et al., 2020; Chung et al., 2021). Thus, we believe that the human alphacoronavirus universal primer set developed in this study is applicable to clinical samples.

Phylogenetic analysis of coronaviruses has been performed using RNA-dependent RNA polymerase (RdRp) protein or spike proteins. However, in the case of the former, genetic distances are not clearly distinguished in some alpha and betacoronaviruses, which can lead to misidentification (Du et al., 2016). Thus, phylogenetic analyses of coronaviruses mainly use the S protein, although low accuracy emerges as an issue when phylogenetic analysis is performed using a single gene with a large mutation rate (Li et al., 2020). When we compared the S protein tree and genome tree, the topologies varied greatly depending on the trees. Remarkably, NL63-related bat coronavirus BtKYNL63-9b was placed within the Non-HCoV group in the S protein amino acid tree but is positioned within the HCoV-like group in the genome tree. Considering that this virus is an obvious Non-HCoV species, the result suggested that the S protein single gene tree would be better in resolution than the whole-genome tree.

## Conclusion

In this study, we developed universal primers that can amplify the entire genome of human alphacoronaviruses, and the experimental conditions were optimized. We successfully amplified 3–5 kb genomic fragments from the genomic RNA of the 229E and NL63 strains using the developed experimental method. The entire genome was sequenced with an error rate of <0.01% regardless of the sequencing platform used. This optimal protocol for human alphacoronavirus genome amplification and sequencing may enable faster diagnosis of mutant alphacoronaviruses in future epidemics.

## Data Availability Statement

The datasets presented in this study can be found in online repositories. The names of the repository/repositories and accession number(s) can be found in the article/Supplementary Material.

## Author Contributions

SC, KWK, and DP performed the experiments. SC and KWK conducted the bioinformatics analyses for sequencing data. SK and HY designed the study and interpreted the data. KBK, S-JK, and CP cultivated the viruses. SC, KWK, SK, and HY were the major contributors in writing the manuscript. All authors contributed to the article and approved the submitted version.

## Funding

This work was supported by the National Research Council of Science & Technology (NST) grant by the Ministry of Science and ICT (MSIT; no. CRC-16-01-KRICT) and by the Technology Innovation Program (Development of rapid molecular diagnostic system for respiratory virus, no. 20012427) funded by the Ministry of Trade, Industry & Energy (MOTIE), Korea.

## Conflict of Interest

The authors declare that the research was conducted in the absence of any commercial or financial relationships that could be construed as a potential conflict of interest.

## Publisher’s Note

All claims expressed in this article are solely those of the authors and do not necessarily represent those of their affiliated organizations, or those of the publisher, the editors and the reviewers. Any product that may be evaluated in this article, or claim that may be made by its manufacturer, is not guaranteed or endorsed by the publisher.

## Supplementary Material

The Supplementary Material for this article can be found online at: https://www.frontiersin.org/articles/10.3389/fmicb.2022.789665/full#supplementary-material

## Footnotes

^1^ www.ViPRbrc.org
