# Supplementary material for: Human Alphacoronavirus Universal Primers for Genome Amplification and Sequencing
Source: Front Microbiol. 2022 Mar 25;13:789665. doi: 10.3389/fmicb.2022.789665 (PMC8990890; doi:10.3389/fmicb.2022.789665)
Supplement: Supplementary file 1 [file Data_Sheet_1.PDF]

Supplementary Figure 1. The analytical pipeline and performance of the sequencing platforms. Genome coverage means the percentage area of the consensus sequence covered by the assembled contig. Accuracy means the proportion of matched nucleotides in the assembled contig compared to the consensus sequence.

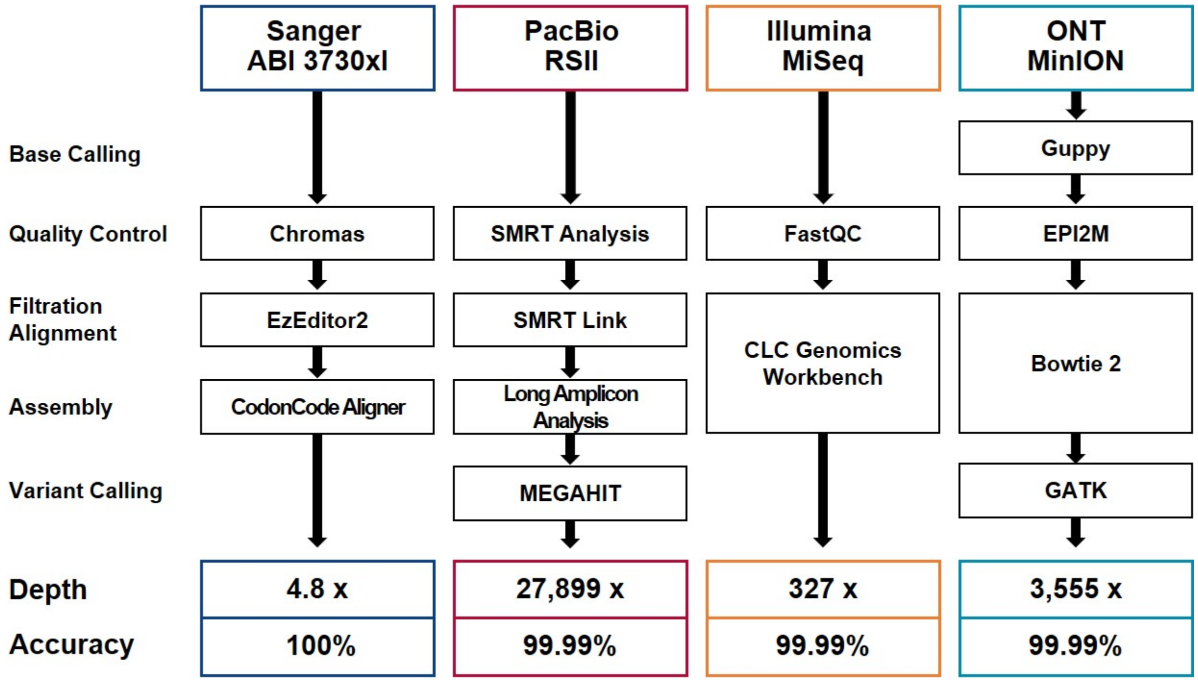

Supplementary Figure 2. Whole genome tree of alphacoronaviruses. The 28 representative genome sequences of 14 subgenera of alphacoronavirus were included in the neighbor-joining tree. Sequences in the 1–43 and >27,270 nt positions (according to the nt positions of the HCoV-229E genome sequence) were excluded from the analysis. HCoV-OC43, a betacoronavirus, was used as an outgroup.

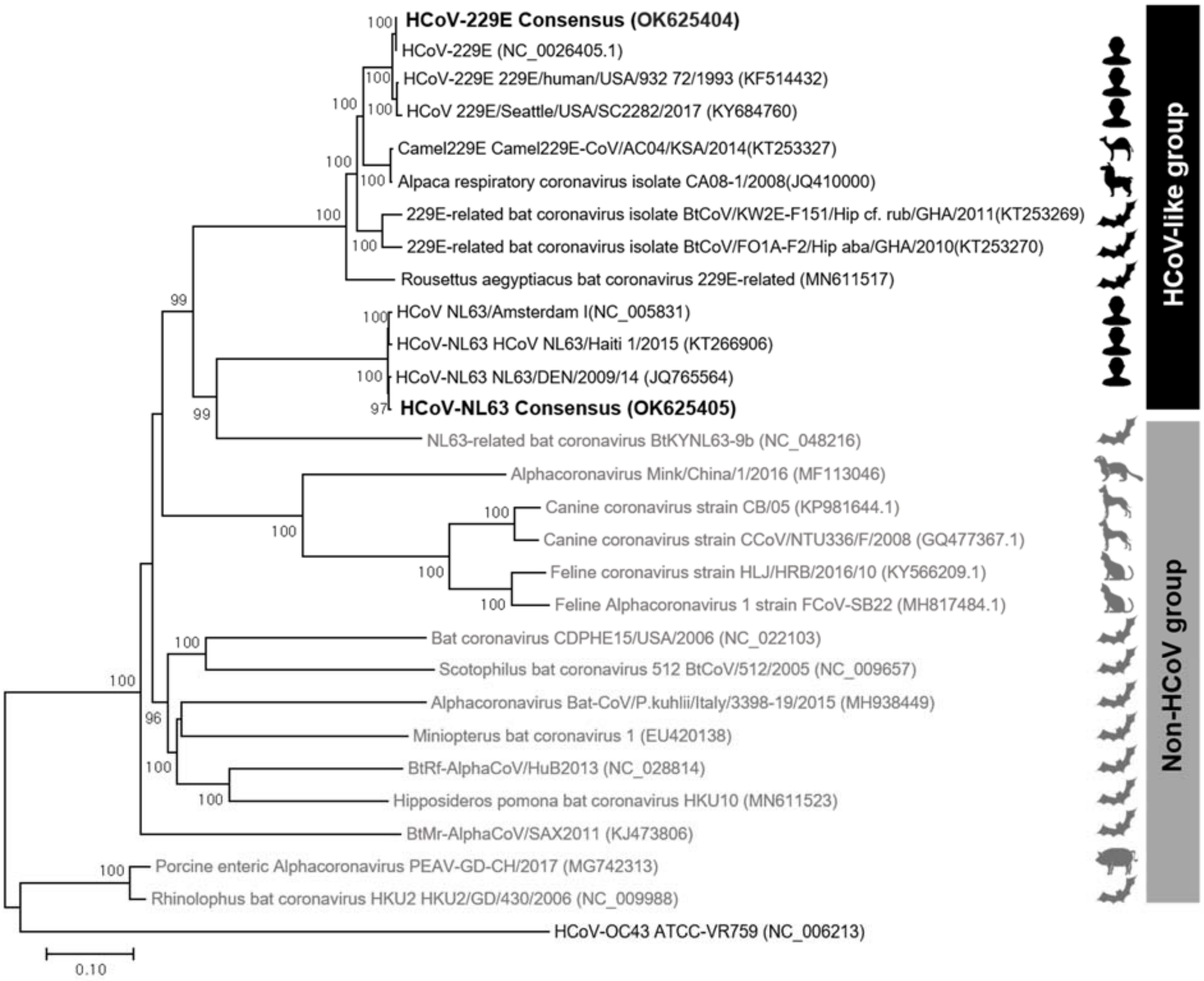

Supplementary Figure 3. Primer mismatches observed in the HCoV-like and non-HCoV clades. Primers (AC01–10) were aligned with the representative genome of the alphacoronavirus subgenus. The observed mismatches between individual primers and genomic sequences are indicated.

			AC01		AC02		AC03		AC04		AC05		AC06		AC07		AC08		AC09		AC10		Total
			F	R	F	R	F	R	F	R	F	R	F	R	F	R	F	R	F	R	F	R	
1	HCoV-229E_Consensus	HCoV-like group	0	0	0	0	0	0	0	0	0	1	0	0	1	0	0	0	0	0	0	0	2
2	HCoV-229E_(NC_002645.1)		0	0	0	0	0	0	0	0	0	0	0	0	0	0	0	0	0	0	0	0	0
3	HCoV-229E_229E/human/USA/932_72/1993_(KF514432)		0	0	0	0	0	0	1	0	0	0	0	0	0	0	0	0	0	0	0	0	1
4	HCoV_229E/Seattle/USA/SC2282/2017_(KY684760)		0	0	0	0	0	0	1	0	0	0	0	0	0	0	0	0	0	0	0	0	1
5	229E-related_bat_coronavirus_isolate_BtCoV/KW2E-F151/Hip_cf._rub/GHA/2011(KT253269)		0	0	0	0	0	0	0	0	0	0	0	0	0	0	0	1	0	0	1	0	2
6	229E-related_bat_coronavirus_isolate_BtCoV/FO1A-F2/Hip_aba/GHA/2010(KT253270)		1	0	0	0	0	0	0	0	0	0	0	0	0	0	0	0	0	0	0	0	1
7	Camel229E_Camel229E-CoV/AC04/KSA/2014(KT253327)		-	0	0	0	0	0	0	0	0	0	0	0	0	0	0	0	0	0	0	0	0
8	Alpaca_respiratory_coronavirus_isolate_CA08-1/2008(JQ410000)		0	0	0	0	0	0	0	0	0	0	0	0	0	0	0	0	0	0	0	0	0
9	Rousettus_aegyptiacus_bat_coronavirus_229E-related_(MN611517)		0	0	0	0	0	2	0	0	1	0	0	2	0	1	2	1	1	0	1	-	11
10	HCoV-NL63_Consensus		0	0	0	0	1	0	0	0	0	0	0	0	0	0	0	0	0	0	0	0	1
11	HCoV_NL63/Amsterdam_I(NC_005831)		0	0	0	0	0	0	0	0	0	0	0	0	0	0	0	0	0	0	0	0	0
12	HCoV-NL63_NL63/DEN/2009/14_(JQ765564)		1	0	0	0	0	0	0	0	0	0	0	0	0	0	0	0	0	0	0	0	1
13	HCoV-NL63_HCoV_NL63/Haiti_1/2015_(KT266906)		0	0	0	0	0	0	0	0	0	0	0	0	0	0	0	0	0	0	0	0	0
14	NL63-related_bat_coronavirus_BtKYNL63-9b_(NC_048216)	Non HCoV-like group	0	1	4	1	4	2	1	1	2	0	1	1	0	1	1	4	2	5	4	1	36
15	Alphacoronavirus_Bat-CoV/P.kuhlii/Italy/3398-19/2015_(MH938449)		18	2	7	4	9	4	4	0	2	4	0	1	4	3	1	9	1	12	9	3	97
16	Alphacoronavirus_Mink/China/1/2016_(MF113046)		0	0	1	8	6	4	8	0	1	2	0	4	2	0	4	6	1	11	6	-	64
17	Bat_coronavirus_CDPHE15/USA/2006_(NC_022103)		5	1	0	6	5	4	6	4	1	2	4	4	2	1	4	6	5	12	6	-	78
18	BtMr-AlphaCoV/SAX2011_(KJ473806)		5	3	4	5	5	7	5	2	1	0	2	2	0	3	2	2	3	6	2	0	59
19	BtRf-AlphaCoV/HuB2013_(NC_028814)		-	0	0	2	9	4	2	1	2	2	1	4	2	3	4	4	3	11	4	6	64
20	Hipposideros_pomona_bat_coronavirus_HKU10_(MN611523)		3	3	3	4	5	3	4	2	3	2	2	0	2	1	0	5	2	11	5	2	62
21	Miniopterus_bat_coronavirus_1_(EU420138)		2	4	3	3	2	5	3	1	2	1	1	0	1	2	0	4	2	13	4	11	64
22	Scotophilus_bat_coronavirus_512_BtCoV/512/2005_(NC_009657)		3	3	3	6	4	4	6	5	1	0	5	3	0	1	3	7	2	4	7	12	79
23	Porcine_enteric_Alphacoronavirus_PEAV-GD-CH/2017_(MG742313)		3	2	4	2	8	7	2	1	3	3	1	4	3	3	4	8	2	5	8	11	84
24	Rhinolophus_bat_coronavirus_HKU2_HKU2/GD/430/2006_(NC_009988)		3	3	3	2	8	7	2	1	2	3	1	4	3	2	4	8	2	4	8	11	81
25	Canine_coronavirus_strain_CB/05_(KP981644.1)		27	3	4	6	6	5	5	2	3	4	2	6	4	4	6	5	2	10	5	1	110
26	Canine_coronavirus_strain_CCoV/NTU336/F/2008_(GQ477367.1)		27	4	5	6	6	5	5	3	3	4	3	7	4	4	7	6	3	9	6	1	118
27	Feline_coronavirus_strain_HLJ/HRB/2016/10_(KY566209.1)		26	0	3	4	6	6	4	4	3	3	4	6	3	4	6	4	3	11	4	1	105
28	Feline_Alphacoronavirus_1_strain_FCoV-SB22_(MH817484.1)		22	0	4	4	5	6	3	3	3	2	3	5	2	4	5	5	3	12	5	1	97
29	HCoV-OC43_ATCC-VR759_(NC_006213)		6	8	4	10	8	10	10	3	4	7	3	8	7	4	8	10	12	15	10	9	156

Supplementary Table 1. Strain-specific primers designed for primer walking and Sanger sequencing in this study.

Name	Sequence
P00001F	ACTTAAGTACCTTATCTAT
P00048F	TTTTGTGTCTACTTTTCTCA
P00478F	TATGGGTCTGCATGGCAAT
P00772F	TATGGGTCTGCATGGCAAT
P00803F	CGTTGTTTGCAACATAATGA
P01491F	GCGTAAGCCCTTAGATTAC
P01538F	GCGTAAGCCCTTAGATTAC
P02807F	AACCTTGCCATTGAAGAGAT
P02776F	AACCTTGCCATTGAAGAGAT
P03530F	TTGTTCGCAAGGCTGAAGA
P04803F	TTGTTCGCAAGGCTGAAGA
P04812F	CTTGTTGGTCCGCTCTTG
P05549F	CTTGTTGGTCCGCTCTTG
P06773F	ATGTGGCGTACTTTGTTAGT
P06819F	GACCCTACACATTTTGACAT
P07531F	TACTTTCGAGAGTGCTTACA
P08534F	TACTTTCGAGAGTGCTTACA
P08561F	ATGGGCCGAGTTGCCAA
P10758F	ATGGGCCGAGTTGCCAA
P10794F	TTAGTGGTGTGCTTAGTGC
P11650F	TGCTTAGTGCTGCTATTAAG
P11918F	GTTGCAAGACTAATGAAAGG
P11977F	GGTGATGCTGAAGACACT
P12036F	GGTGATGCTGAAGACACT
P12249F	TGTTCTAAGAGTGCTAGAC
P12759F	TCAATGGTGTGCAACGTTC
P12843F	AGCTTATGACTTGGCGTG
P14232F	TGCAGGTGCTAAGGTTGTT
P14757F	TGCAGGTGCTAAGGTTGTT
P14794F	GTTGCTGCAATGTCAGGT
P15012F	GTTGCTGCAATGTCAGGT
P16793F	CTTAATTGTGCATTAGGTGC
P16835F	TAGGTGCTACTATGCATGG
P16998F	TTTCACAGACTAACATGCAC
P18817F	GGTTCTGTAAGTGCACATTA
P18848F	GTGTTAGTCCAGCCGAATT
P19463F	GCTCCGACGTTTGGACAT
P19980F	GGAAGTTAAGGATAATGATGG
P19989F	GGAAGTTAAGGATAATGATGG
P20152F	GTTAAATGGGAACATGACTC
P20713F	TGTTATAGACACACCTACTG
P20771F	ACAATTTAAGGAGAGGTGCT
P22608F	AGGCTATTACTTGTACCATTG
P22736F	TGCTGTTGCTAAGCATGATT
P24191F	CACCATGATGGATTTGTGC
P24756F	TCGTTACAACAGACCTACTA
P24798F	ACAAGCTCGCGTAGCATAT
P26524F	ATGGGATGGGACTATCCTA
P26676F	CCGATGTTGATGATCCTAAA

Name	Sequence
P00478R	GCTCTATCACACTTAGGATA
P00501R	TAAGATCATAGCCGACAACA
P00772R	AGCACACAACACTATCATCA
P00803R	CAGCAATGTAACCAAGTCC
P01491R	AGTCTTAGTGATGTCATCC
P01538R	AGTCTTAGTGATGTCATCC
P01983R	AACAGAATTACGGTTTAACG
P02776R	CAAACACAAACTCACCAAC
P03530R	CACAGCCTTGCTCCATG
P05522R	AGCCTGGCAGCTACATAAT
P05549R	GTTCTGTCAGACATAATACAG
P07504R	TTCTCCAGGTGGTGCAC
P07531R	TTGTCCCTTGCGTAACAGA
P08534R	CAGTACCACATTGGCAATG
P09417R	GAATACTGAGAGGTGGTGT
P09456R	CTTATATGTGGCGACAACAC
P11461R	CTTATATGTGGCGACAACAC
P11650R	ATGCTGCCACAAACTCCT
P13848R	GATCATTAAGATAAGTCACAG
P13881R	GATCATTAAGATAAGTCACAG
P14300R	CAGTAACACTAAAATCAGCG
P14335R	CACAATCACCAGTAACACTA
P14375R	ACTAACAAAGTACGCCACAT
P15012R	TTAATAGCAATCGAACCTCC
P15800R	AATGCAAAGTCGGAGGGT
P15859R	AATGCAAAGTCGGAGGGT
P17484R	AACTCCTCACAACACCATC
P17520R	AGTGAAGTGTGATGTGTCAA
P17683R	GGCATAAACAGCAACGTATT
P18848R	TGTAAATACTCAACCTGGAC
P19421R	AAGCAATTCAACACAGCGC
P19463R	ATCTCACTAACATCTGCAGA
P01983R	GTCCACAGTGCCAAGTC
P20713R	TTGAGCACAAGCCAAGTC
P21591R	GAGCAAGATTAGGTTGTCG
P21615R	GGAATACGTGGTTCAAACAT
P22343R	ACAGTGCCATCTTTCACGT
P22420R	ACAGTGCCATCTTTCACGT
P22502R	TGATCATCCACTAGCTTAAG
P23498R	AGCACCACACACCAGAG
P23558R	ACCCGTACAATTGTCATTTG
P24191R	GAACCTGAACACCTGAAG
P24999R	TATAGTAGTGCTCGGCAC
P25461R	AAGACCACAGTGTTGCCT
P25508R	AAGACCACAGTGTTGCCT
P26524R	ACTTACCCAAGTGTGGATG
P27264R	TTTTTTTGTGTATCCATATCG
PolyT	TTTTTTTTTTTTTTTTTTT

Supplementary Table 2. Primer mismatches observed in the HCoV-like clade. Primers (AC01–10) were aligned with the genomes of the alphacoronaviruses. The observed mismatches between individual primers and genomic sequences are indicated.

#	Strain	Genome sequence	Accession number	AC01		AC02		AC03		AC04		AC05		AC06		AC07		AC08		AC09		AC10		Total
				F	R	F	R	F	R	F	R	F	R	F	R	F	R	F	R	F	R	F	R	
1	229E	0304	JX503061	0	0	0	0	0	0	0	0	0	0	0	0	0	0	0	0	0	0	0	0	0
2	229E	229E/China/01/2009	MW532106	0	0	0	0	0	0	0	0	0	0	0	0	0	0	0	0	0	0	0	0	0
3	229E	229E/China/02/2009	MW532107	0	0	0	0	0	0	0	0	0	0	0	0	0	0	0	0	0	0	0	0	0
4	229E	229E/China/03/2017	MW532103	0	0	0	0	0	0	0	0	0	0	0	0	0	0	0	0	0	0	0	-	0
5	229E	229E/China/04/2017	MW532104	0	0	0	0	0	0	0	0	0	0	0	0	0	0	0	0	0	0	0	0	0
6	229E	229E/China/05/2017	MW532105	0	0	0	0	0	0	0	0	0	0	0	0	0	0	0	0	0	0	0	-	0
7	229E	229E/Haiti_1/2016	MF542265	0	0	0	0	0	0	0	0	0	0	0	0	0	0	0	0	0	0	0	0	0
8	229E	229E/human/USA/932_72/1993	KF514432	0	0	0	0	0	0	0	0	0	0	0	0	0	0	0	0	0	0	0	0	0
9	229E	229E/human/USA/933_40/1993	KF514433	0	0	0	0	0	0	0	0	0	0	0	0	0	0	0	0	0	0	0	0	0
10	229E	229E/human/USA/933_50/1993	KF514430	0	0	0	0	0	0	0	0	0	0	0	0	0	0	0	0	0	0	0	0	0
11	229E	229E/UF_1/2016	KY996417	0	0	0	0	0	0	0	0	0	0	0	0	0	0	0	0	0	0	0	0	0
12	229E	229E_S220	MW202340	0	0	0	0	0	0	0	0	0	0	0	0	0	0	0	0	0	0	0	0	0
13	NL63	Amsterdam_057	DQ445911	0	0	0	0	0	0	0	0	0	0	0	0	0	0	0	0	0	0	0	0	0
14	NL63	Amsterdam_496	DQ445912	1	0	0	0	0	0	0	0	0	0	0	0	0	0	0	0	0	0	0	0	1
15	NL63	Amsterdam_I	NC005831	0	0	0	0	0	0	0	0	0	0	0	0	0	0	0	0	0	0	0	0	0
16	229E	**BtCoV/AT1A_F1/Hip_aba/GHA/2010**	KT253272	0	0	0	0	0	0	0	0	0	0	0	0	0	0	0	10	0	0	-	0	**10**
17	229E	BtCoV/FO1A_F2/Hip_aba/GHA/2010	KT253270	1	0	0	0	0	0	0	0	0	0	0	0	0	0	0	0	0	0	0	0	1
18	229E	BtCoV/KW2E_F151/Hip_cf._rub/GHA/2011	KT253269	0	0	0	0	0	0	0	0	0	0	0	0	0	0	0	1	0	0	1	0	2
19	229E	**BtCoV/KW2E_F56/Hip_cf._rub/GHA/2011**	KT253271	0	0	0	0	0	1	0	0	0	0	0	0	0	0	0	5	1	0	5	0	**12**
20	229E	**BtKY229E_1**	KY073747	0	0	0	0	0	0	0	0	0	0	0	0	0	1	0	2	1	0	2	-	**6**
21	229E	**BtKY229E_8**	KY07374	0	0	1	0	1	0	0	0	0	0	0	1	0	1	1	3	2	0	3	-	**13**
22	229E	CA08_1/2008	JQ410000	0	0	0	0	0	0	0	0	0	0	0	0	0	0	0	0	0	0	0	0	0
23	229E	camel/Abu_Dhabi/B38	MF593473	0	0	0	0	0	0	0	0	0	0	0	0	0	0	0	0	0	0	0	0	0
24	229E	camel/Riyadh/Ry141/2015	KT368907	0	0	0	0	0	0	0	0	0	0	0	0	0	0	0	0	0	0	0	0	0
25	229E	Camel229E_CoV/AC04/KSA/2014	KT253327	0	0	0	0	0	0	0	0	0	0	0	0	0	0	0	0	0	0	0	0	0
26	229E	Camel229E_CoV/JC49/KSA/2014	KT253325	0	0	0	0	0	0	0	0	0	0	0	0	0	0	0	0	0	0	0	2	2
27	229E	Camel229E_CoV/JC50/KSA/2014	KT253324	0	0	0	0	0	0	0	0	0	0	0	0	0	0	0	0	0	0	0	0	0
28	229E	Camel229E_CoV/JC50_HUH7/KSA/2014	KT253328	0	0	0	0	0	0	0	0	0	0	0	0	0	0	0	0	0	0	0	0	0
29	229E	Camel229E_CoV/JC52/KSA/2014	KT253326	0	0	0	0	0	0	0	0	0	0	0	0	0	0	0	0	0	0	0	2	2
30	229E	**Camel229E_CoV/KCSP1/KEN/2015**	KU291449	0	0	0	0	0	0	0	0	0	0	0	0	0	0	0	2	0	0	2	2	**6**
31	NL63	CBJ_037	JX104161	0	0	0	0	0	0	0	0	0	0	0	0	0	0	0	0	0	0	0	0	0
32	NL63	CBJ123	JX524171	0	0	0	0	0	0	0	0	0	0	0	0	0	0	0	0	0	0	0	0	0
33	NL63	ChinaGD01	MK334046	1	0	0	0	0	0	0	0	0	0	0	0	0	0	0	0	0	0	0	0	1
34	NL63	ChinaGD02	MK334043	0	0	0	0	0	0	0	0	0	0	0	0	0	0	0	0	0	0	0	0	0
35	NL63	ChinaGD03	MK334044	0	0	0	0	0	0	0	0	0	0	0	0	0	0	0	0	0	0	0	0	0
36	NL63	ChinaGD04	MK334047	2	0	0	0	0	0	0	0	0	0	0	0	0	0	0	0	0	0	0	0	2
37	NL63	ChinaGD05	MK334045	0	0	0	0	0	1	0	0	0	0	0	0	0	0	0	0	0	0	0	0	1
38	NL63	CN0601/14	MG772808	1	0	0	0	1	0	0	0	0	0	0	0	0	0	0	0	0	0	0	0	2
39	229E	Fukushima_H219_2018	LC654455	0	0	0	0	0	0	0	0	0	0	0	0	0	0	0	0	0	0	0	0	0
40	229E	Fukushima_H829_2020	LC654445	0	0	0	0	0	0	0	0	0	0	0	0	0	0	0	0	0	0	0	0	0
41	229E	Fukushima_H832_2020	LC654446	0	0	0	0	0	0	0	0	0	0	0	0	0	0	0	0	0	0	0	0	0
42	229E	HCoV_229E/BN1/GER/2015	KU291448	0	0	0	0	0	0	0	0	0	0	0	0	0	0	0	0	0	0	0	2	2
43	229E	HCoV_229E/Seattle/USA/SC0865/2019	MN306046	0	0	0	0	0	0	0	0	0	0	0	0	0	0	0	0	0	0	0	0	0
44	229E	HCoV_229E/Seattle/USA/SC1073/2016	KY369913	0	0	0	0	0	0	0	0	0	0	0	0	0	0	0	0	0	0	0	0	0
45	229E	HCoV_229E/Seattle/USA/SC1143/2016	KY369910	0	0	0	0	0	0	0	0	0	0	0	0	0	0	0	0	0	0	0	0	0
46	229E	HCoV_229E/Seattle/USA/SC1212/2016	KY369911	0	0	0	0	0	0	0	0	0	0	0	0	0	0	0	0	0	0	0	0	0
47	229E	HCoV_229E/Seattle/USA/SC2282/2017	KY684760	0	0	0	0	0	0	0	0	0	0	0	0	0	0	0	0	0	0	0	0	0
48	229E	HCoV_229E/Seattle/USA/SC2872/2015	KY967357	0	0	0	0	0	0	0	0	0	0	0	0	0	0	0	0	0	0	0	0	0
49	229E	HCoV_229E/Seattle/USA/SC3112/2015	KY983587	0	0	0	0	0	0	0	0	0	0	0	0	0	0	0	0	0	0	0	0	0
50	229E	HCoV_229E/Seattle/USA/SC379/2016	KY621348	0	1	0	0	0	0	0	0	0	0	0	0	0	0	0	0	0	0	0	0	1
51	229E	HCoV_229E/Seattle/USA/SC399/2016	KY674914	0	0	0	0	0	0	0	0	0	0	0	0	0	0	0	0	0	0	0	0	0
52	229E	HCoV_229E/Seattle/USA/SC579/2016	KY369908	0	0	0	0	0	0	0	0	0	0	0	0	0	0	0	0	0	0	0	0	0
53	229E	HCoV_229E/Seattle/USA/SC677/2016	KY369909	0	0	0	0	0	0	0	0	0	0	0	0	0	0	0	0	0	0	0	0	0
54	229E	HCoV_229E/Seattle/USA/SC9724/2018	MN369046	0	0	0	0	0	0	0	0	0	0	0	0	0	0	0	0	1	0	0	0	1
55	229E	HCoV_229E/Seattle/USA/SC9731/2016	KY369912	0	0	0	0	0	0	0	0	0	0	0	0	0	0	0	0	0	0	0	0	0
56	229E	HCoV_229E/Seattle/USA/SC9773/2016	KY369914	1	0	0	0	0	0	0	0	0	0	0	0	0	0	0	0	0	0	0	0	1
57	229E	HCoV_229E/USA/ACRI_0244/2017	MT438697	0	2	0	0	0	0	0	0	0	0	0	0	0	0	0	0	0	0	0	0	2
58	229E	HCoV_229E/USA/ACRI_0246/2017	MT438699	0	1	0	0	0	0	0	0	0	0	0	0	0	0	0	0	0	0	0	-	1
59	229E	HCoV_229E/USA/ACRI_0256/2017	MT438698	1	0	0	0	0	0	0	0	0	0	0	0	0	0	0	0	0	0	0	0	1
60	229E	HCoV_229E/USA/UAMS_DID_0108/2017	MT438700	1	1	0	0	0	0	0	0	0	0	0	0	0	0	0	0	0	0	0	0	2
61	229E	HCoV_229E/USA/UNM_0186/2016	MT438696	2	0	0	0	0	0	0	0	0	0	0	0	0	0	0	0	0	0	0	0	2
62	NL63	HCoV_NL63/Haiti_1/2015	KT266906	0	0	0	0	0	0	0	0	0	0	0	0	0	0	0	0	0	0	0	0	0
63	NL63	HCoV_NL63/Seattle/USA/SC0179/2018	MN306018	0	0	0	0	0	0	0	0	0	0	0	0	0	0	0	0	0	0	0	0	0
64	NL63	HCoV_NL63/Seattle/USA/SC0768/2019	MN306040	0	0	0	0	-	0	0	0	0	0	0	0	0	0	0	0	-	0	0	0	0
65	NL63	HCoV_NL63/Seattle/USA/SC2940/2015	KY983586	1	0	0	0	0	0	0	0	0	0	0	0	0	0	0	0	0	0	0	0	1
66	229E	HK20_42	MT797634	0	2	0	0	0	0	0	0	0	0	0	0	0	0	0	0	0	0	0	0	2
67	229E	Human coronavirus 229E	NC002645	0	0	0	0	0	0	0	0	0	0	0	0	0	0	0	0	0	0	0	0	0
68	229E	Human coronavirus 229E isolate 0349	JX503060	0	0	0	0	0	0	0	0	0	0	0	0	0	0	0	0	0	0	0	0	0
69	NL63	Human coronavirus NL63	DJ009246)	0	0	0	0	0	0	0	0	0	0	0	0	0	0	0	0	0	0	0	0	0
70	NL63	Human group 1 coronavirus	AY518894	1	0	0	0	0	0	0	0	0	0	0	0	0	0	0	0	0	0	0	0	1
71	NL63	Kilifi_HH_0511_01_Jun_2010	MG428703	0	0	0	0	0	0	0	0	0	0	0	0	0	0	0	0	0	0	0	0	0
72	NL63	Kilifi_HH_0512_04_Jun_2010	MG428701	0	0	0	0	0	0	0	0	0	0	0	0	0	0	0	0	0	0	0	0	0
73	NL63	Kilifi_HH_0522_21_May_2010	MG428705	0	0	0	0	0	0	0	0	0	0	0	0	0	0	0	0	0	0	0	-	0
74	NL63	Kilifi_HH_3807_11_May_2010	MG428702	-	-	0	0	0	0	0	0	0	0	0	0	0	0	0	0	0	0	0	0	0
75	NL63	Kilifi_HH_3808_24_May_2010	MG428706	0	0	0	0	0	0	0	0	0	0	0	0	0	0	0	0	0	0	0	0	0
76	NL63	Kilifi_HH_5402_20_May_2010	MG428704	0	0	0	0	0	0	0	0	0	0	0	0	0	0	0	0	0	0	0	0	0
77	NL63	Kilifi_HH_5709_19_May_2010	MG428699	0	0	0	0	0	0	0	0	0	0	0	0	0	0	0	0	0	0	0	0	0
78	NL63	N06_1144B	KY554967	1	0	0	0	0	0	0	0	0	0	0	0	0	0	0	0	0	0	0	0	1
79	NL63	N07_185B	KY554968	1	0	0	0	0	0	0	0	0	0	0	0	0	0	0	0	0	0	0	0	1
80	NL63	N07_196B	KY554969	0	0	0	0	0	0	0	0	0	0	0	0	0	0	0	0	0	0	0	0	0
81	NL63	N07_262B	KY829118	0	0	0	0	0	0	0	0	0	0	0	0	0	0	0	0	0	0	0	0	0
82	NL63	N07_324B_182X	KY554970	1	0	0	0	0	0	0	0	0	0	0	0	0	0	0	0	0	0	0	0	1
83	NL63	N07_468B_176X	KY554971	0	0	0	0	0	0	0	0	0	0	0	0	0	0	0	0	0	0	0	-	0
84	NL63	N07_64B	KY674916	1	0	0	0	0	0	0	0	0	0	0	0	0	0	0	0	0	0	0	0	1
85	NL63	N07_6B	KY674915	0	0	0	0	0	0	0	0	0	0	0	0	0	0	0	0	0	0	0	0	0
86	229E	N08_434B	KY674919	0	0	0	0	0	0	0	0	0	0	0	0	0	0	0	0	1	0	0	0	1
87	NL63	NL63/DEN/2005/1062	JQ765573	0	0	0	0	0	0	0	0	0	0	0	0	0	0	0	0	0	0	0	0	0
88	NL63	NL63/DEN/2005/1120	JQ900260	0	0	0	-	1	0	-	0	0	0	0	-	0	0	-	0	0	0	0	0	1
89	NL63	NL63/DEN/2005/1862	JQ765574	0	0	0	0	0	0	0	0	0	0	0	0	0	0	0	0	0	0	0	0	0
90	NL63	NL63/DEN/2005/1876	JQ765575	0	0	0	0	0	0	0	0	0	0	0	0	0	0	0	0	0	0	0	0	0
91	NL63	NL63/DEN/2005/193	JQ765568	0	0	0	0	0	0	0	0	0	0	0	0	0	0	0	0	0	0	0	0	0
92	NL63	NL63/DEN/2005/232	JQ765569	1	0	0	0	0	0	0	0	0	0	0	0	0	0	0	0	0	0	0	0	1
93	NL63	NL63/DEN/2005/235	JQ765570	1	0	0	0	0	0	0	0	0	0	0	0	0	0	0	0	0	0	0	0	1
94	NL63	NL63/DEN/2005/271	JQ765571	0	0	0	0	0	0	0	0	0	0	0	0	0	0	0	0	0	0	0	0	0
95	NL63	NL63/DEN/2005/291	JQ900258	0	0	-	-	0	0	-	0	0	0	0	0	0	0	0	0	0	0	0	0	0
96	NL63	NL63/DEN/2005/347	JQ765572	0	0	0	0	0	0	0	0	0	0	0	0	0	0	0	0	0	0	0	0	0
97	NL63	NL63/DEN/2005/449	JQ900259	0	0	0	0	0	0	0	0	0	0	0	0	0	0	0	0	0	0	0	0	0
98	NL63	NL63/DEN/2008/16	JQ765566	1	0	0	0	0	0	0	0	0	0	0	0	0	0	0	0	0	0	0	0	1
99	NL63	NL63/DEN/2009/14	JQ765564	1	0	0	0	0	0	0	0	0	0	0	0	0	0	0	0	0	0	0	0	1
100	NL63	NL63/DEN/2009/15	JQ765565	1	0	0	0	0	0	0	0	0	0	0	0	0	0	0	0	0	0	0	0	1
101	NL63	NL63/DEN/2009/20	JQ765567	1	0	0	0	0	0	0	0	0	0	0	0	0	0	0	0	0	0	0	0	1
102	NL63	NL63/DEN/2009/22	JQ900256	1	0	0	0	0	0	0	0	0	0	0	0	0	0	0	0	0	0	0	0	1
103	NL63	NL63/DEN/2009/31	JQ900257	0	0	0	0	0	0	0	0	0	0	0	0	0	0	0	0	0	0	0	0	0
104	NL63	NL63/DEN/2009/6	JQ900255	1	0	0	0	0	0	0	0	0	0	0	0	0	0	0	0	0	0	0	0	1
105	NL63	NL63/DEN/2009/9	JQ765563	1	0	0	0	0	0	0	0	0	0	0	0	0	0	0	0	0	0	0	0	1
106	NL63	NL63/human/USA/0111_25/2001	KF530112	0	0	0	0	0	0	0	0	0	0	0	0	0	0	0	0	0	0	0	-	0
107	NL63	NL63/human/USA/012_31/2001	KF530105	0	0	0	0	0	0	0	0	0	0	0	0	0	0	0	0	0	0	0	-	0
108	NL63	NL63/human/USA/838_9/1983	KF530110	0	0	0	0	0	0	0	0	0	0	0	0	0	0	0	0	0	0	0	-	0
109	NL63	NL63/human/USA/8712_17/1987	KF530106	0	0	0	0	0	0	0	0	0	0	0	0	0	0	0	0	0	0	0	-	0
110	NL63	NL63/human/USA/891_4/1989	KF530114	0	0	0	0	0	0	0	0	0	0	0	0	0	0	0	0	0	0	0	-	0
111	NL63	NL63/human/USA/891_6/1989	KF530108	0	0	0	0	0	0	0	0	0	0	0	0	0	0	0	0	0	0	0	-	0
112	NL63	NL63/human/USA/901_24/1990	KF530111	0	0	0	0	0	0	0	0	0	0	0	0	0	0	0	0	0	0	0	-	0
113	NL63	NL63/human/USA/903_28/1990	KF530109	0	0	0	0	0	0	0	0	0	0	0	0	0	0	0	0	0	0	0	-	0
114	NL63	NL63/human/USA/904_20/1990	KF530104	0	0	0	0	0	0	0	0	0	0	0	0	0	0	0	0	0	0	0	-	0
115	NL63	NL63/human/USA/905_25/1990	KF530113	0	0	0	0	0	0	0	0	0	0	0	0	0	0	0	0	0	0	0	-	0
116	NL63	NL63/human/USA/911_56/1991	KF530107	0	0	0	0	0	0	0	0	0	0	0	0	0	0	0	0	0	0	0	-	0
117	NL63	NL63/RPTEC/2004	JX504050	0	0	0	0	0	0	0	0	0	0	0	0	0	0	0	0	0	0	0	0	0
118	NL63	NL63/UF_1/2015	KT381875	1	0	0	0	0	0	0	0	0	0	0	0	0	0	0	0	0	0	0	0	1
119	NL63	NL63/UF_2/2015	KX179500	1	0	0	0	0	0	0	0	0	0	0	0	0	0	0	0	0	0	0	0	1
120	NL63	NL63/UF_2/2015	KU521535	1	0	0	0	0	0	0	0	0	0	0	0	0	0	0	0	0	0	0	0	1
121	NL63	NL63_S170	MW202337	0	0	0	0	0	0	0	0	0	0	0	0	0	0	0	0	0	0	0	0	0
122	NL63	Sequence 55 from Patent EP1553169	CS124012	0	0	0	0	0	0	0	0	0	0	0	0	0	0	0	0	0	0	0	0	0
123	NL63	Tokyo/SGH_15/2017	LC488390	1	0	0	0	0	0	0	0	0	0	0	0	0	0	0	0	0	0	0	0	1
124	NL63	Tokyo/SGH_18/2018 (LC488389)	LC488389	1	0	0	0	0	0	0	0	0	0	0	0	0	0	0	0	0	0	0	0	1
