# Supplementary figures and images for: Human Alphacoronavirus Universal Primers for Genome Amplification and Sequencing
Source: Front Microbiol. 2022 Mar 25;13:789665. doi: 10.3389/fmicb.2022.789665 (PMC8990890; doi:10.3389/fmicb.2022.789665)

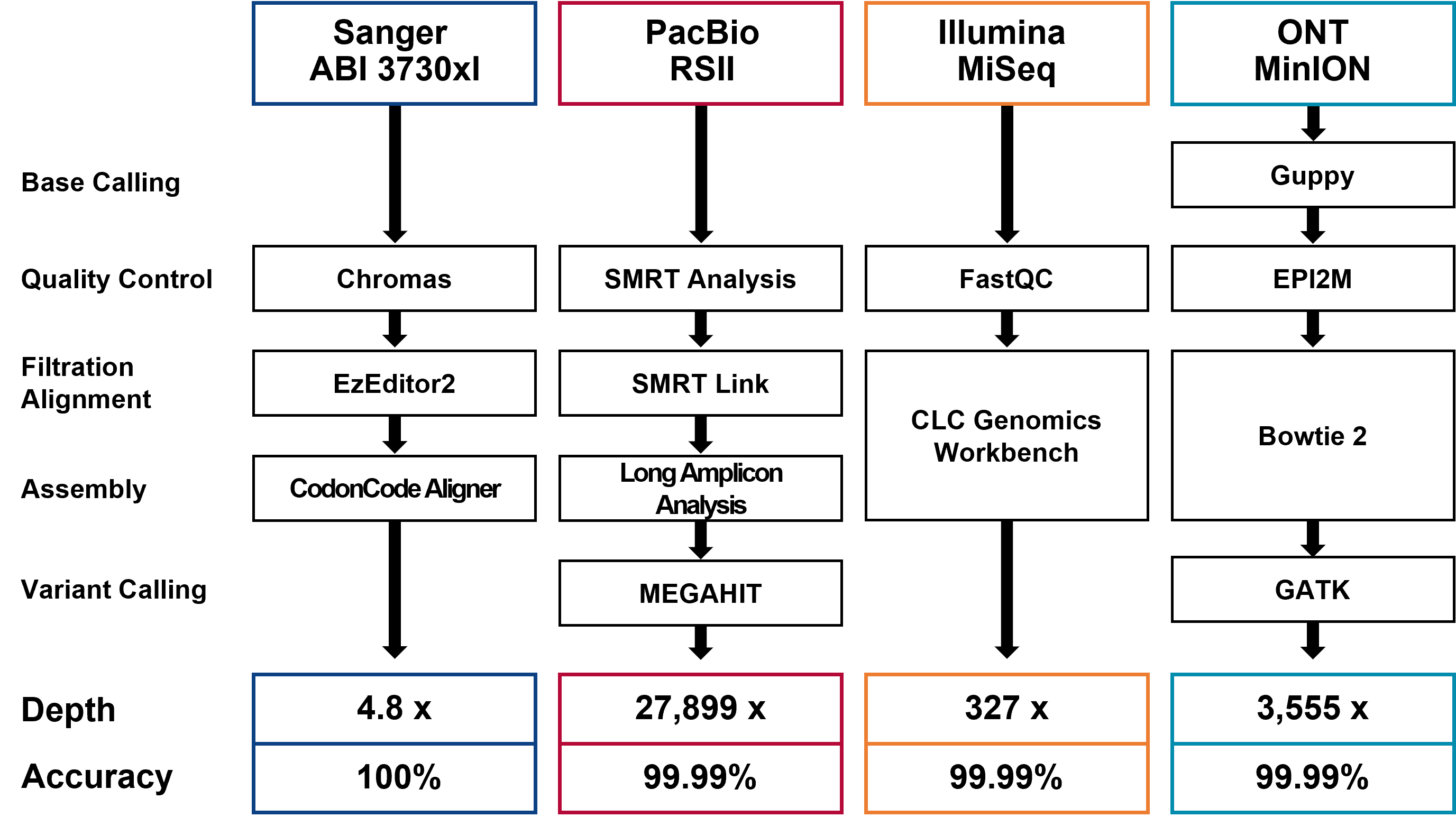

Supplement: Supplementary file 2 [file Image_1.TIF]

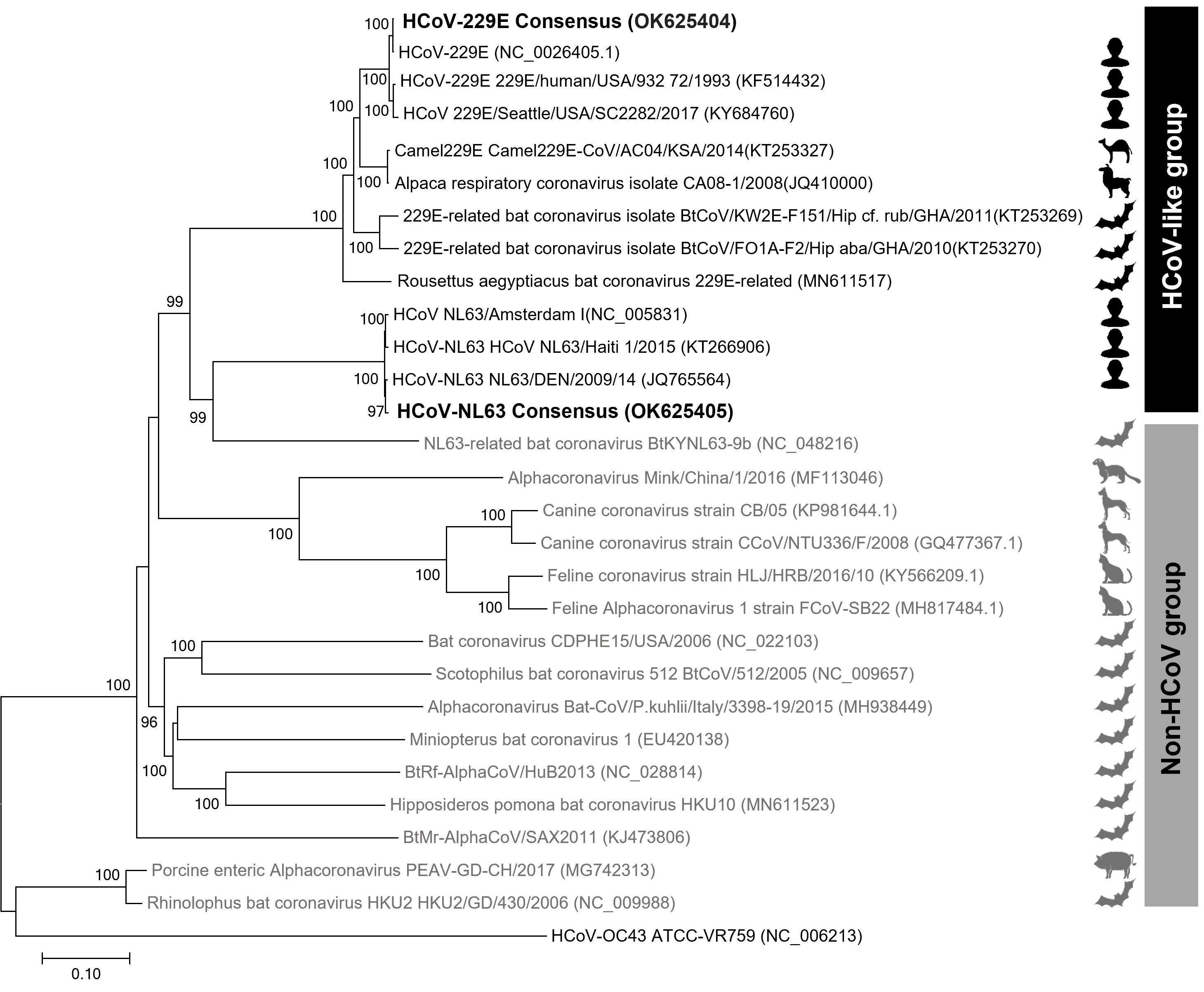

Supplement: Supplementary file 3 [file Image_2.TIF]

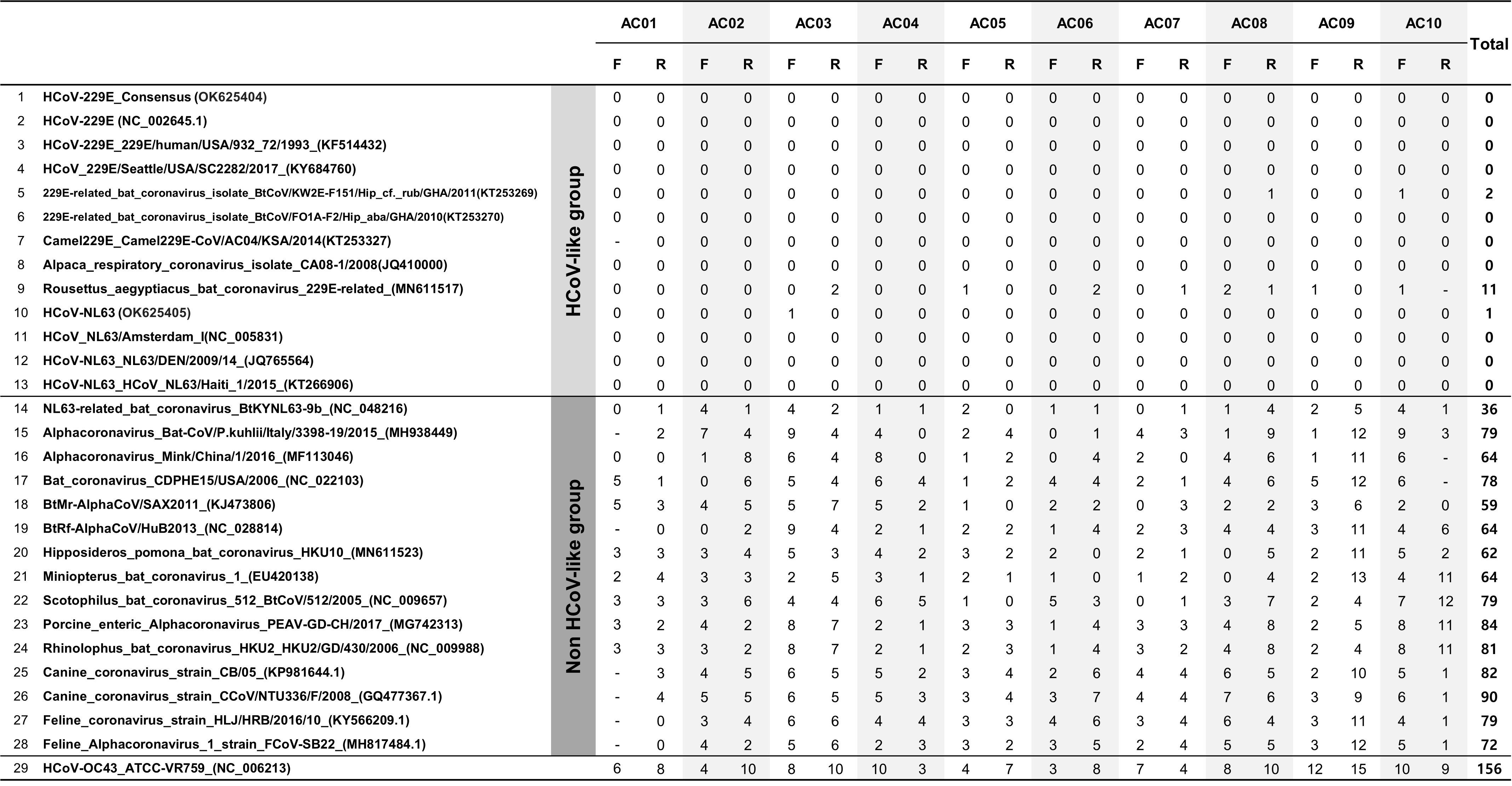

Supplement: Supplementary file 4 [file Image_3.TIF]
